# Different Functions of Human Scavenger Receptors BI and BII Overexpressed in a Murine Abdominal Sepsis Model

**DOI:** 10.3390/biom16050670

**Published:** 2026-05-01

**Authors:** Naoki Hayase, Tatyana G. Vishnyakova, Irina N. Baranova, Alexander V. Bocharov, Xuzhen Hu, Amy P. Patterson, Peter S. T. Yuen, Thomas L. Eggerman, Robert A. Star

**Affiliations:** 1 Renal Diagnostics and Therapeutics Unit, National Institute of Diabetes, Digestive and Kidney Diseases, National Institutes of Health, Bethesda, MD 20892-1268, USA; naoki.hayase@nih.gov (N.H.); susanhu989@gmail.com (X.H.); starr@niddk.nih.gov (R.A.S.); 2 Department of Laboratory Medicine, Clinical Center, National Institutes of Health, Bethesda, MD 20892, USA; vishnyakova@hotmail.com (T.G.V.); ibaranova1@gmail.com (I.N.B.); abocharov@cc.nih.gov (A.V.B.); amy.patterson@nih.gov (A.P.P.); eggermant@extra.niddk.nih.gov (T.L.E.); 3 Office of the Director, Division of Program Coordination, Planning and Strategic Initiatives, National Institutes of Health, Bethesda, MD 20892, USA; 4 Division of Diabetes, Endocrinology and Metabolic Diseases, National Institute of Diabetes and Digestive and Kidney Diseases, National Institutes of Health, Bethesda, MD 20892, USA

**Keywords:** adrenal gland, cecal ligation and puncture, class B scavenger receptor BI, class B scavenger receptor BII, corticosterone, high density lipoprotein, kidney, liver, sepsis

## Abstract

Class B scavenger receptor BI splice variants (SR-BI) and BII (SR-BII) internalize lipoproteins but also bind and internalize bacteria. Their individual roles in sepsis are unknown. We overexpressed human SR-BI or BII in transgenic mice, primarily in the liver, but also in the kidney and in bone marrow-derived macrophages, and then performed cecal ligation and puncture (CLP) surgery. SR-BI and BII transgenic mice had significantly worse survival compared to WT mice. Twenty-four hours after CLP, liver injury markers and histological damage were elevated in both SR-BI and BII transgenic mice, whereas kidney damage was similar. Systemic inflammatory cytokines were markedly increased in SR-BI and BII transgenic mice; parallel increases were seen in liver mRNA expression, but not in the kidney. The highest degree of neutrophil infiltration was observed in the liver of SR-BI. Human SR-BI and BII dramatically decreased bacterial accumulation in the liver. Green fluorescent protein-labeled *E. coli* were efficiently phagocytosed in hepatic macrophages of SR-BI and BII transgenic mice; phagocytosis was more prominent in SR-BII transgenic mice. Finally, human SR-BI overexpression reduced systemic HDL-C levels, eliminated adrenal cortex lipid droplets, and dampened the systemic increase of corticosterone after CLP. Supplementation with glucocorticoid and mineralocorticoid improved survival in SR-BI but not in SR-BII transgenic mice after CLP. In summary, our findings suggest human SR-BI and BII overexpression contributes to higher mortality after CLP by different mechanisms: excessive inflammatory response due to adrenal insufficiency (SR-BI) or hyperactive phagocytosis (SR-BII) in the liver.

## 1. Introduction

Sepsis is a life-threatening condition characterized by an excessive host immune response to infection. Sepsis-induced immune alterations have been associated with both poor short- and long-term outcomes [1]. These initial alterations are thought to be driven, in part, by the activation of pattern-recognition receptors (PRRs), which initiate inflammatory responses upon sensing pathogen-associated molecular patterns (PAMPs), leading to immune system hyperactivation [2]. However, to date, the immunoadjuvant treatments that function by blocking PRRs (e.g., Eritoran and Resatorvid, both of which are Toll-like receptor-4 inhibitors) have failed to improve 28-day mortality in septic patients [3,4]. A comprehensive understanding of PRR functions, not only in the innate immune system but also in other biological systems, including the metabolic and endocrine systems, is essential for developing more targeted PRR-based therapies and minimizing off-target effects.

The class B scavenger receptor BI (SR-BI) and its splice variant BII (SR-BII) are predominantly expressed in the liver, steroidogenic tissues, parenchymal epithelial cells of many organs, and phagocytic cells [5,6,7,8,9]. These receptors are well known for their roles in lipid metabolism, mediating both the selective uptake of high-density lipoprotein cholesterol (HDL-C) and the efflux of cholesterol from peripheral tissues via HDL [5,10]. Our earlier in vitro studies demonstrated that both SR-BI and SR-BII function as PRRs by binding to and facilitating the internalization of bacteria, as well as initiating inflammatory responses through interactions with bacterial components such as lipopolysaccharide (LPS), lipoteichoic acid, and the cytosolic protein GroEL [11,12].

To evaluate the roles of SR-BI and SR-BII in in vivo animal models of sepsis, SR-BI/BII knockout (KO) mice were initially studied, but it was not possible to differentiate between them. SR-BI/BII KO mice had elevated systemic inflammatory cytokines and higher mortality after cecal ligation and puncture (CLP) surgery than wild-type (WT) controls. These findings suggest that SR-BI and BII may play a protective role against CLP-induced sepsis, although it is unclear if the two splice variants have similar or different roles. Septic SR-BI/BII KO mice had a profound defect in corticosterone production (adrenal insufficiency) [13], which complicated the interpretation of the experimental results. The same study further reported that treatment with corticosterone alone (glucocorticoid only) did not improve survival in SR-BI/BII KO mice [13]. In contrast, when using mice treated with glucocorticoid and mineralocorticoid before CLP surgery [14], we demonstrated that SR-BI/BII KO attenuated proinflammatory responses and improved survival. Thus, the survival outcomes of SR-BI/SR-BII KO mice after CLP could depend on the details of adrenal cortical hormone replacement.

Our previous in vitro studies also suggested SR-B receptors can promote pathogen internalization, escape from lysosomal killing, cytosolic proliferation, and amplified inflammation [12,14,15,16]. Therefore, based on both in vitro and in vivo results with steroid replacement, we hypothesized that SR-BI and BII may exert net detrimental effects in sepsis.

The SR-BI/BII KO mice developed adrenal insufficiency during sepsis probably because the lack of SR-BI/BII prevented HDL-C uptake into the adrenal gland and decreased both gluco-and mineralocorticoid responses, which then worsened sepsis. To better understand the role of SR-Bs in sepsis, we generated SR-BI and SR-BII transgenic mice, with the transgene overexpressed mainly in the liver. The transgenic mice are easier to generate than SR-BI- and SR-BII-specific knock-out mouse lines. We assumed that we could then evaluate the roles of SR-BI and SR-BII in innate immunity during sepsis with less impact from confounding adrenal insufficiency [17]. This study aimed to elucidate the pathogenetic roles of human SR-BI and SR-BII in the CLP-induced sepsis model fully treated with fluid, antibiotics, and analgesics—similar to the treatments critically ill patients would receive in an intensive care unit.

## 2. Materials and Methods

### 2.1. Animals

All animal experiments were conducted according to the National Institutes of Health Guide for the Care and Use of Laboratory Animals (U.S. Department of Health and Human Services Public Health Services, National Institutes of Health, Bethesda, Maryland; publication No. 85–23, 1985) and were approved by the NIDDK Animal Care and Use Committee (K100-KDB) on 12 January 2024. We obtained 8- to 12-week-old male C57BL/J mice with an average weight of 20 to 25 g from the Jackson Laboratory (Bar Harbor, ME). We previously developed human SR-BI and human SR-BII transgenic mice using the liver-specific expression vector pLiv-11, which contains the human apoE promoter, on a C57BL/6J background [17]. The hepatic control region enabled transgenes to be expressed predominantly in hepatocytes [18]. A TaqMan PCR assay confirmed that human SR-BI and SR-BII expression was highest in the liver of SR-BI and SR-BII transgenic mice, respectively. The expression of human SR-B was also detectable in the kidney, lung, and spleen, but at 100- to 1000-fold lower extent than in the liver [17]. Moreover, we reported that human SR-BI and BII were highly expressed in bone marrow-derived macrophages in the respective transgenic mice [17]. The mice were kept under specific pathogen-free conditions in a 12 h light/dark cycle with free access to chow and water and were acclimatized for at least 1 week before use. All surgeries were performed on a heated operating table under isoflurane anesthesia during the daytime. An extended-release formulation of buprenorphine (0.5 mg/kg Buprenorphine ER, SR Veterinary Technologies, Windsor, CO) was subcutaneously administered for analgesia immediately before the surgeries and every 72 h for survival studies.

### 2.2. Total RNA Isolation and Quantitative PCR Analysis of Proinflammatory Cytokines in Livers and Kidneys

Collected liver and kidney samples were homogenized in TRIzol reagent using a Precellys 24 homogenizer (Bertin Technologies, Montigny-le-Bretonneux, France). RNA was isolated with the PureLink RNA mini kit (Thermo Fisher Scientific, Waltham, MA, USA) after DNase treatment. Complementary DNA was synthesized from total RNA with a TaqMan reverse transcriptase reagent kit (Thermo Fisher Scientific). Quantitative reverse transcription polymerase chain reaction (RT-PCR) was performed with a StepOne real-time PCR system (Applied Biosystems, Foster City, CA, USA). The mRNA levels were examined using TaqMan gene expression assays (Applied Biosystems, Foster City, CA, USA) and TaqMan primers for chemokine (C-X-F motif) ligand 1 (CXCL1, Mm04207460_m1), tumor necrosis factor-α (TNF-α, Mm00443258_m1), interleukin-6 (IL-6, Mm00446190_m1), and glyceraldehyde-3-phosphate dehydrogenase (GAPDH, Mm03302249_m1). Relative levels of gene expression were calculated by the comparative cycle threshold (CT) method with GAPDH genes as a reference gene. All gene expression results were analyzed using the 2^−ΔΔCT^ method and are presented as normalized fold changes compared to the averaged results for corresponding sham-operated controls. The expression of human SR-BI and BII transgenes was analyzed using the following custom primers: for human SR-BI, forward, 5′-GGTCCCTGTCATCTGCCAA-3′, and reverse, 5′-CTCCTTATCCTTTGAGCCCTTT-3′; and for human SR-BII, forward, 5′-TCCTGAGGACACCGTGAGC-3′, and reverse, 5′-GAGGCTCAGGCTGTGG-3′ [19]. The results were expressed relative to the average hepatic expression level.

### 2.3. Cecal Ligation and Puncture

Cecal ligation and puncture (CLP) was conducted as reported previously [20]. Briefly, the cecum was ligated with (4-0 silk) 10 mm from the cecal tip. The cecum was punctured twice with a 21-gauge needle and gently squeezed to express a ~1 mm column of fecal content. Prewarmed 0.9% saline (40 mL/kg) was injected intraperitoneally. Sham-operated mice were subjected to identical procedures except for the ligation and puncture of the cecum. Treatment with fluid and antibiotics was started at 6 h after surgery with a subcutaneous injection of imipenem/cilastatin (14 mg/kg Primaxin, Merck, Whitehouse Station, NJ, USA) in 40 mL/kg of 0.9% saline. In acute studies, animals were euthanized 24 h after surgery to collect blood and organ specimens. In the 7-day survival studies, the treatment was continued every 12 h with imipenem/cilastatin (7 mg/kg) in 40 mL/kg of 0.6% saline. Additional doses of Buprenorphine ER were administered every 72 h after surgery. Sepsis severity was determined according to Murine Sepsis Score with minor modifications [21]. Animals exceeding a predefined threshold of the severity score (≥15) were euthanized and counted as non-survivors.

### 2.4. E. coli Injection Model

*E. coli* K12 and green fluorescent protein (GFP)-labeled *E. coli* (ATCC, Manassas, VA, USA) were grown, harvested, washed twice with phosphate-buffered saline (PBS), and diluted to 1.6 × 10^7^ c.f.u./mL in PBS. To simulate invading bacteria in the CLP sepsis model, mice were intraperitoneally injected with 600 μL of bacterial suspension and after 8 h were perfused with 20 mL PBS. Then the liver and kidneys were harvested.

### 2.5. Immunofluorescence Analyses of Mouse Livers and Kidneys

Harvested livers and kidneys were frozen in Tissue-Tek OCT compound (Sakura Finetek, Torrance, CA, USA) and cryosectioned at a thickness of 5 μm. The sections were fixed with 4% paraformaldehyde (PFA) for 20 min and blocked with 1% bovine serum albumin and 1% goat serum albumin in phosphate-buffered saline for 1 h at room temperature. Subsequently, the tissues were incubated overnight at 4 °C in 2 μg/mL rabbit anti-mouse F4/80 antibody (Bio-Rad, Hercules, CA, USA) as a primary antibody. After washing, the sections were incubated with Alexa 647–goat anti-rabbit IgG (2 μg/mL) for 1 h at room temperature (Thermo Fisher Scientific). DNA was stained with 2 μg/mL Hoechst 33342 (Thermo Fisher Scientific). Confocal images were acquired using a Zeiss LSM 700 confocal microscope (Zeiss, Jena, Germany). Randomly selected nonoverlapping fields (*n* = 3–6) were captured for each liver and kidney section using oil immersion objectives with ×20 or ×63 magnification. The total number of GFP-labeled bacteria was determined, and the proportion of bacteria localized inside F4/80-positive cells was then calculated. In a separate experiment, F4/80-positive cells were counted in each field and then normalized by the total nucleus count. The above-mentioned imaging analysis was conducted in a blinded manner using Fiji v. 2.17.0 (National Institutes of Health, Bethesda, MD, USA).

### 2.6. Histological Examination of Organ Damage and Neutrophils

Liver and kidney specimens were fixed with 10% formalin and embedded in paraffin. The 4-μm sections were stained with periodic acid-Schiff (PAS) reagent. The extent of tubular injury was evaluated by a semiquantitative scoring system in 10 non-overlapping cortical fields under ×400 magnification and averaged per mouse. The percentage of tubules that displayed cellular necrosis, loss of brush border, cast formation, vacuolization, and tubule dilation was scored as follows: (0) none, (1) 1–25%, (2) 26–50%, (3) 51–75%, and (4) 76–100%. The percentage of glycogen-positive area in the whole surface area in the liver was measured using Fiji v. 2.17.0 (National Institutes of Health) in 4–7 images taken for each mouse under ×200 magnification to assess liver function. For identifying neutrophils, the naphthol AS-D chloroacetate esterase kit (Sigma-Aldrich, St. Louis, MO, USA) was used for the liver and kidneys. Neutrophils infiltrating the liver and kidneys were counted in 7–12 non-overlapping fields under ×400 magnification. The evaluator was blinded to the origin of the samples until after histological quantitation.

### 2.7. Immunohistochemical Analysis for Green Fluorescent Protein-Labeled E. coli

Liver and kidney sections were deparaffinized and incubated in sodium citrate buffer (10 mM sodium citrate, 0.05% Tween 20, pH 6.0) for 20 min at 98 °C using a microwave. Endogenous peroxidase activity was blocked with 0.3% hydrogen peroxide in methyl alcohol for 15 min. After blocking with goat serum, the specimens were incubated overnight at 4 °C with 5 µg/mL rabbit anti-GFP antibody (ab290; Abcam, Cambridge, MA, USA). Horseradish peroxidase-conjugated goat anti-rabbit antibody (Agilent Dako, Santa Clara, CA, USA) was applied to sections and incubated for 1 h at room temperature. Sections were developed using 3,3′-diaminobenzidine tetrahydrochloride (Sigma-Aldrich), and then counterstained with hematoxylin.

### 2.8. Oil Red O Staining

Adrenal glands and liver were embedded in Tissue-Tek OCT compounds (Sakura Finetek) and cryosectioned at a thickness of 5 μm. The sections were fixed in 4% paraformaldehyde for 10 min. After incubating with 60% isopropanol for 2 min, the slides were stained with a freshly prepared Oil Red O working solution for 15 min. Then, the slides were rinsed with 60% isopropanol, followed by hematoxylin counterstaining for 15 s. The slides were rinsed with distilled water before mounting a coverslip onto the slides with warmed glycerol gelatin. The lipid droplet area in the adrenal cortex was measured with Fiji 2.17.0 (National Institutes of Health) under ×400 magnification and was expressed as a percentage of the adrenal cortex area.

### 2.9. Measurement of Blood Chemistry, Cytokines, Vascular Endothelial Growth Factor, High-Density Lipoprotein Cholesterol, and Corticosterone

Blood chemistry (serum blood urea nitrogen, aspartate aminotransferase, and alanine aminotransferase) was measured using commercial assays (VRL Diagnostics, Gaithersburg, MD, USA). Cystatin C, interleukin-6 (IL-6), tumor necrosis factor-alpha (TNF-α), vascular endothelial growth factor (VEGF), high-density lipoprotein cholesterol (HDL-C) and corticosterone were measured using the corresponding mouse enzyme-linked immunosorbent assay (ELISA) kits according to the manufacturer’s instructions. IL-6, TNF-α, and VEGF ELISA kits were obtained from R&D Systems (Minneapolis, MN, USA). HDL-C, corticosterone, and Cystatin C ELISA kits were purchased from AFG Scientific (Northbrook, IL), Enzo Life Sciences (Farmington, NY, USA), and BioVendor (Candler, NC, USA), respectively.

### 2.10. Bacterial Count in Blood, Peritoneal Lavage Fluid, and Organs

Anesthetized mice were washed with 70% ethanol under sterile conditions at 24 h after CLP surgery. Peritoneal lavage was performed with 5 mL sterile PBS. The lavage fluid was collected using an 18-gauge needle before blood was drawn by cardiac puncture. The liver and kidneys were harvested and homogenized. Serial dilutions of blood, peritoneal lavage fluid, and organ homogenate were plated on 5% sheep blood agar plates (BD, Franklin Lakes, NJ, USA). The plates were incubated at 37 °C for 24 h and bacterial colonies were counted. A value of 0.5 colony-forming unit was assigned to the samples that did not have detectable bacterial colonies.

### 2.11. Glucocorticoid and Mineralocorticoid Supplementation

A glucocorticoid and mineralocorticoid (GM) cocktail of 6α-methylprednisolone and fludrocortisone acetate (Sigma-Aldrich) was prepared at a similar potency as described in a previous study [22]. Mice were subcutaneously supplemented with vehicle (20 mL/kg of 3% dimethyl sulfoxide diluted with 0.9% saline) or a cocktail of 1.6 mg/kg 6α-methylprednisolone and 40 μg/kg fludrocortisone acetate in vehicle immediately after CLP surgery. The treatment with the vehicle or GM cocktail was continued daily at half doses for the following 4 days.

### 2.12. Study Design

We used three experimental groups: wild-type (WT), SR-BI, and SR-BII for 7-day survival analysis, microbiological evaluation, and immunofluorescence assays using GFP-labeled *E. coli*. Assessment of biochemistry, histological organ damage, inflammatory cytokines, VEGF, HDL-C, corticosterone, and lipid droplets was conducted using six experimental groups: sham-operated or baseline WT, SR-BI, and SR-BII groups and CLP-injured WT, SR-BI, and SR-BII groups. Additionally, two groups were employed to investigate the impact of GM supplementation on septic SR-BI and BII transgenic mice: GM-treated and vehicle-treated groups. The experimental unit was a single animal.

### 2.13. Randomization and Blinding

The investigators were blinded to the genotype and treatment group in all experiments. A third-party allocated a random number to each mouse cage ID using the RAND function (Excel version 16, Microsoft, Redmond, WA, USA). The IDs were then sorted from smallest to largest allocation number to create a randomly ordered ID list. Letter labels were assigned to the cage IDs in this order. The third party replaced the original cage card with the corresponding letter card. The surgeon (NH) performed CLP surgery, bacterial infusion, or a sham operation in alphabetical order of the letter cards. The WT, SR-BI, and BII transgenic mice were indistinguishable by appearance. The third-party randomized SR-BI and BII mice into the vehicle and GM treatments using the RAND function and prepared the drug for each mouse according to the allocated group. Unblinding occurred after all analyses were completed.

### 2.14. Sample Size Calculation

In a pilot study, we compared the 7-day survival rates of WT (*n* = 7), SR-BI transgenic (*n* = 8), and SR-BII transgenic mice (*n* = 9) after CLP surgery. The respective survival rates were 28.6%, 0.0%, and 0.0%. To detect a 29% difference in the survival rate in both comparisons—WT vs. SR-BI transgenic and WT vs. SR-BII transgenic—we estimated the effect size as 1.137 and calculated a sample size of 14 mice per group, assuming a two-sided α level of 0.05 and power of 0.80.

### 2.15. Statistical Analysis

The Shapiro–Wilk test was used to assess the normality of distribution of all continuous variables. The data are expressed as means ± SD when normally distributed and as medians (interquartile ranges) when not normally distributed. For normally distributed data, the Student’s *t*-test was applied to analyze any significant difference between groups. Otherwise, the Wilcoxon rank-sum test was used as a nonparametric test. The survival rates were represented using Kaplan–Meier survival curves and compared using a log-rank test. Multiple pairwise comparisons were conducted using a one-way ANOVA followed by the Holm–Šídák test for parametric values, and a lognormal one-way ANOVA followed by Holm–Šídák test or the Kruskal–Wallis test followed by the Dunn’s test for nonparametric values. Repeated measures analysis was performed using a two-way ANOVA with the Tukey–Kramer test. A two-tailed *p* value of less than 0.05 was considered statistically significant for all tests. All calculations were conducted using GraphPad Prism 10 software (GraphPad Software Inc., La Jolla, CA, USA).

## 3. Results

### 3.1. Overexpression of Human SR-B Transgenes Worsened the Survival and Sepsis Severity of Septic Mice

**Figure 1:**
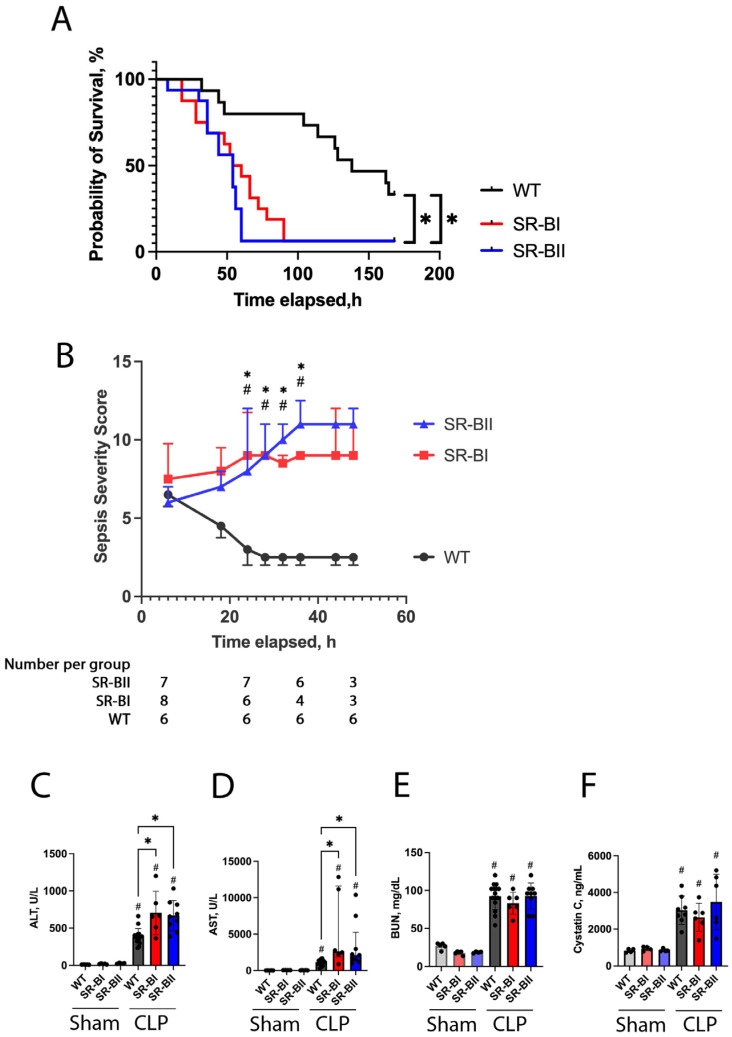
Kaplan–Meier survival curves, sepsis severity scores, circulating hepatic and renal injury markers after cecal ligation and puncture surgery. (**A**) A seven-day survival study was conducted for wild-type (WT) and human class B scavenger receptor BI (SR-BI) and BII (SR-BII) transgenic mice following cecal ligation and puncture (CLP) surgery. Kaplan–Meier survival curves are shown. SR-BI and SR-BII transgenic mice had significantly worse survival compared to WT mice (SR-BI (red) vs. WT (black), 6.3% vs. 33.3%, *p* = 0.001; SR-BII (blue) vs. WT (black), 6.3% vs. 33.3%, *p* = 0.001; *n* = 15 in the WT and *n* = 16 in the SR-BI and SR-BII groups; *n* = 47 total). * *p* < 0.05 versus respective control. (**B**) Longitudinal changes in sepsis severity scores were assessed until the number of remaining animals in either experimental group was reduced to three following CLP surgery. The number of remaining mice in each group at 6, 24, 36, and 48 h is shown under the graph. The data are represented as means ± SD. The comparison between the groups at each time point was performed by Tukey multiple comparison test after a two-way ANOVA test. # *p* < 0.05 between SR-BI and WT, * *p* < 0.05 between SR-BII and WT. (**C**–**F**) Blood was collected at 24 h after CLP or sham operation. Serum alanine aminotransferase (**C**, ALT), aspartate aminotransferase (**D**, AST), blood urea nitrogen (**E**, BUN), and Cystatin C (**F**) levels were measured and compared between the six experimental groups (ALT, AST, and BUN: sham WT, *n* = 5; sham SR-BI, *n* = 5; sham SR-BII, *n* = 4; CLP WT, *n* = 15; CLP SR-BI, *n* = 6; CLP SR-BII, *n* = 9; *n* = 44 total, Cystatin C: sham WT, *n* = 4; sham SR-BI, *n* = 4; sham SR-BII, *n* = 4; CLP WT, *n* = 8; CLP SR-BI, *n* = 6; CLP SR-BII, *n* = 6; *n* = 32 total). ALT, BUN, and Cystatin C data are shown as means ± SD due to their normal distribution. The data of AST are represented as medians (interquartile ranges) due to their skewed distribution. # *p* < 0.05 versus sham, * *p* < 0.05 between WT, SR-BI and SR-BII after CLP or sham.

We conducted a 7-day survival study to examine whether overexpressed human SR-BI or BII could affect the survival of septic mice after CLP surgery. Both human SR-BI and SR-BII transgenic mice had a significantly lower survival rate than WT mice (SR-BI vs. WT, 6.3% vs. 33.3%, *p* = 0.001; SR-BII vs. WT, 6.3% vs. 33.3%, *p* = 0.001). The survival rates of those transgenic mice plummeted to 6.3% within 96 h after CLP, while that of WT mice decreased more gradually and was still 80% at 96 h. Additionally, SR-BI transgenic mice had a comparable survival rate to SR-BII transgenic mice (Figure 1A). Collectively, human SR-BI and SR-BII overexpression resulted in significantly lower 7-day survival of CLP-induced septic mice.

The sepsis severity score of SR-BI and BII transgenic mice was significantly higher than that of WT mice at 24 h after CLP (Figure 1B). The survival curves began to diverge at 24 h. More than 20% of the SR-BI transgenic septic mice died or were euthanized after 24 h (Figure 1A). Therefore, we chose 24 h after CLP as the best timepoint to study underlying mechanisms.

### 3.2. Overexpression of Human SR-B Transgenes Exacerbated Sepsis-Induced Liver Injury

Next, we asked why overexpressed human SR-BI and SR-BII increased the mortality of septic mice. Our previous study confirmed higher mRNA expression and protein production of human SR-B in liver and kidney of SR-B transgenic mice compared to WT mice [17]. Intraperitoneal injection of lipopolysaccharide caused more severe histological damage and immune response in the liver and kidney of SR-B transgenic mice [17]. Therefore, we focused on the liver and kidney, hypothesizing that organ damage could be involved in higher mortality during CLP sepsis.

CLP sepsis caused the elevation of liver and kidney injury markers (ALT, AST, and BUN) in WT and SR-B transgenic mice at 24 h. ALT and AST were significantly increased in SR-BI (75% and 127% increase in ALT and AST, respectively) and SR-BII transgenic mice (73% and 84% increase in ALT and AST, respectively) vs. WT mice (Figure 1C,D). In contrast, the BUN and Cystatin C levels were similar across the experimental groups (Figure 1E,F).

**Figure 2:**
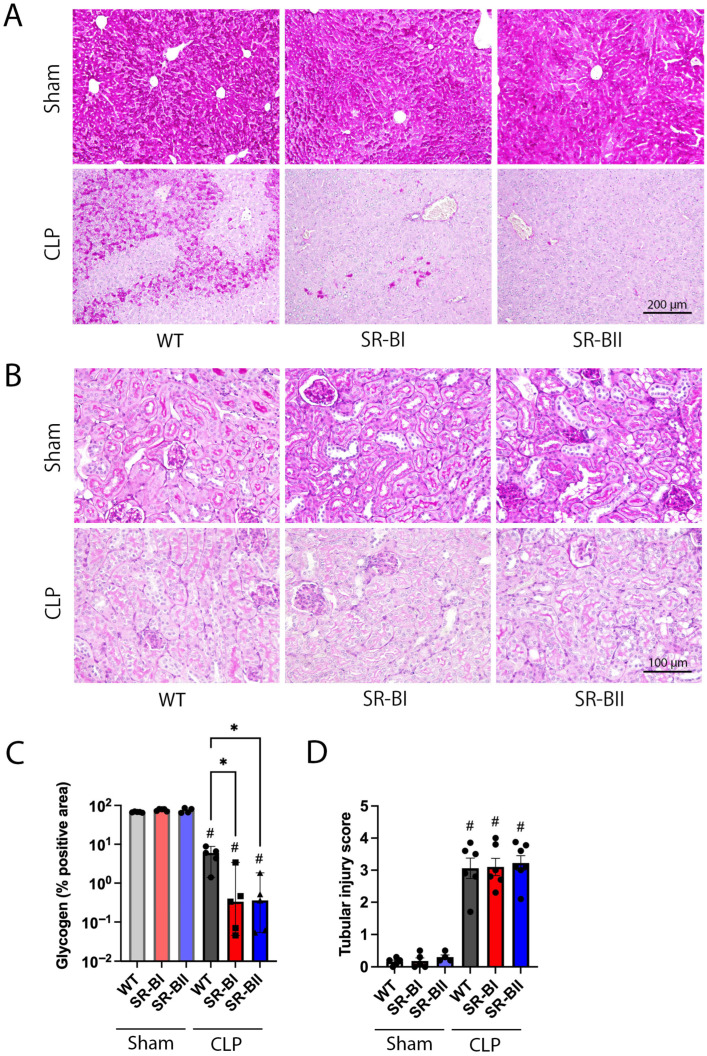
Liver and kidney histological damage at 24 h after cecal ligation and puncture surgery. The liver and kidneys were harvested from wild-type (WT) and human class B scavenger receptor BI (SR-BI) and BII (SR-BII) transgenic mice at 24 h after cecal ligation and puncture (CLP) surgery. (**A**) Representative images of Periodic acid-Shiff (PAS) staining of liver sections are shown. The dark pink area represents liver glycogen, which was significantly reduced after CLP. Bar = 200 µm. (**B**) Representative images of PAS staining in the renal cortex are shown. CLP induced distinct kidney damage, characterized by vacuolar degeneration and blush border loss. Bar = 100 µm. (**C**) The percentage of glycogen-rich area in the whole tissue area was measured using Fiji and compared among the experimental groups (sham WT, *n* = 5; sham SR-BI, *n* = 5; sham SR-BII, *n* = 4; CLP WT, SR-BI, and SR-BII, *n* = 5 per group; *n* = 29 total). The data are represented as medians (interquartile ranges). # *p* < 0.05 versus sham, * *p* < 0.05 between WT, SR-BI and SR-BII after CLP or sham. (**D**) The extent of kidney damage was assessed using tubular injury scores and compared across the experimental groups (sham WT, *n* = 5; sham SR-BI, *n* = 5; sham SR-BII, *n* = 4; CLP WT, *n* = 6; SR-BI, *n* = 6; and SR-BII *n* = 7 per group; CLP SR-BII, *n* = 7; *n* = 33 total). The data are shown as means ± SD. # *p* < 0.05 versus sham.

We then evaluated the histological liver and kidney damage at 24 h after CLP. As expected, CLP sepsis decreased liver glycogen storage represented by changes from deep red/pink to pale pink PAS staining. Liver glycogen was additionally and significantly decreased in SR-BI and SR-BII transgenic mice (84% and 90% reduction in SR-BI and SR-BII, respectively, vs. WT septic mice, Figure 2A,C). All three experimental groups developed sepsis-induced kidney tubular damage as characterized by increased vacuolization and loss of brush border to a similar extent (Figure 2B,D).

Both human SR-BI and SR-BII transgenic mice developed significantly more liver injury compared to WT mice during CLP sepsis despite similar levels of kidney injury.

### 3.3. Human SR-B Enhanced Systemic and Hepatic Proinflammatory Response; Neutrophil Infiltration Was Prominent in the Livers of SR-BI Transgenic Mice

**Figure 3:**
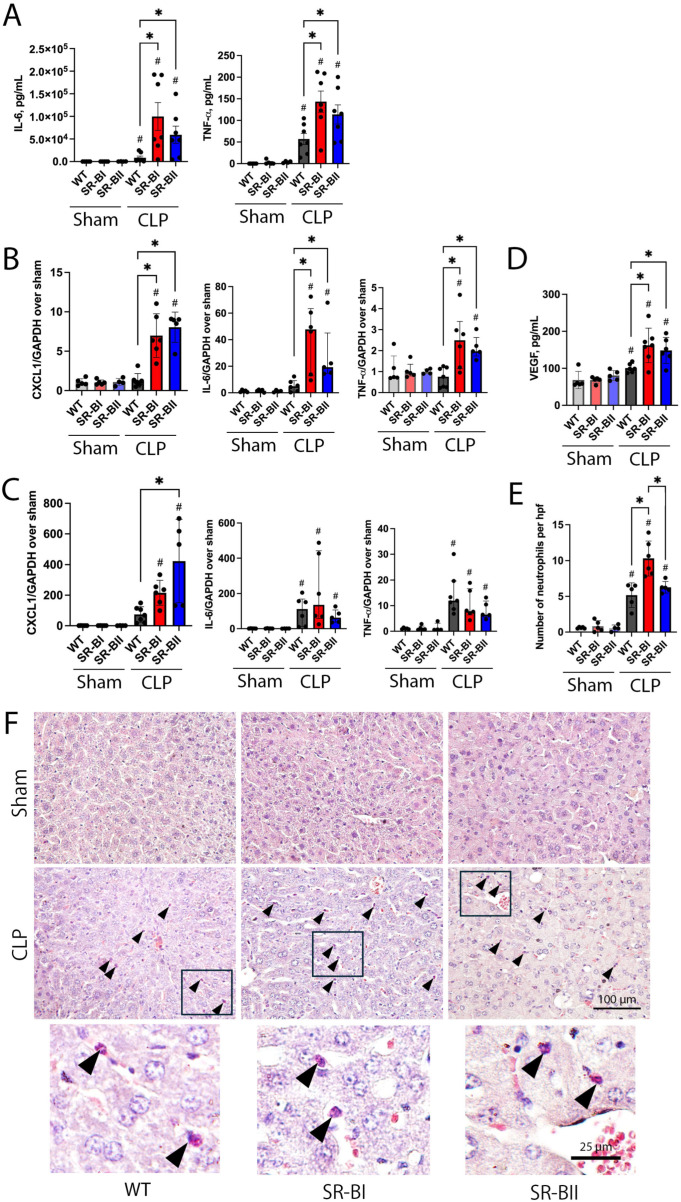
Inflammatory cytokines, vascular endothelial growth factor, and neutrophil infiltration at 24 h after cecal ligation and puncture. The blood, liver, and kidneys were collected from wild-type (WT) and human class B scavenger receptor BI (SR-BI) and BII (SR-BII) transgenic mice at 24 h after cecal ligation and puncture (CLP) surgery. (**A**) Plasma levels of interleukin-6 (IL-6), tumor necrosis factor-α (TNF-α) were assessed and compared among the experimental groups (sham WT, *n* = 5; sham SR-BI, *n* = 5; sham SR-BII, *n* = 4; CLP WT, SR-BI, and SR-BII, *n* = 7 per group; *n* = 35 total). The data are shown as means ± SD. # *p* < 0.05 versus sham, * *p* < 0.05 between WT, SR-BI, and SR-BII after CLP or sham. The mRNA expression levels of chemokine (C-X-F motif) ligand 1 (CXCL1), interleukin-6 (IL-6), and tumor necrosis factor-α (TNF-α) in the liver (**B**) and kidney (**C**) were evaluated and normalized with glyceraldehyde-3-phosphate dehydrogenase (GAPDH) in corresponding experimental groups for the respective organs (sham WT, *n* = 5; sham SR-BI, *n* = 5; sham SR-BII, *n* = 4; CLP WT, *n* = 7; CLP SR-BI, *n* = 6 and SR-BII *n* = 5; *n* = 32 total). The CXCL1 expression levels are expressed as means ± SD due to their normal distribution, whereas IL-6 and TNF-α levels are expressed as medians (interquartile ranges) due to their skewed distribution. # *p* < 0.05 versus sham, * *p* < 0.05 between WT, SR-BI and SR-BII after CLP or sham. (**D**) Plasma vascular endothelial growth factor (VEGF) was measured and compared among the experimental groups (sham WT, SR-BI, and SR-BII, *n* = 5 per group; CLP WT, *n* = 6; CLP SR-BI, *n* = 7 and SR-BII *n* = 6; *n* = 34 total). The data are shown as means ± SD. # *p* < 0.05 versus sham, * *p* < 0.05 between WT, SR-BI, and SR-BII after CLP or sham. (**E**) The number of neutrophils per high-power field (hpf) was compared among the experimental groups (sham WT and SR-BI, *n* = 5 per group; sham SR-BII, *n* = 4; CLP WT, *n* = 5; CLP SR-BI, *n* = 6; CLP SR-BII, *n* = 5; *n* = 30 total). The data are shown as means ± SD. # *p* < 0.05 versus sham, * *p* < 0.05 between WT, SR-BI, and SR-BII after CLP or sham. (**F**) Representative images of naphthol AS-D chloroacetate esterase staining in the liver are exhibited. The arrowheads denote infiltrating neutrophils. Black boxes in the middle row represent the boundaries of the magnified insets in the bottom row. Bar = 100 µm, insets: bar = 25 µm.

Our previous in vivo study using a LPS injection model reported that the production of proinflammatory cytokines and chemokines mediated through the mitogen-activated protein kinase (MAPK) signaling pathway might be a driver of organ injury in human SR-B transgenic mice with endotoxemia [17]. Thus, we evaluated systemic and organ levels of proinflammatory cytokines using ELISA and quantitative RT-PCR. As expected, plasma IL-6 and TNF-α levels were dramatically increased after CLP. Of note, those levels were more prominent in septic SR-B transgenic mice (IL-6: 28- and 12-fold higher in SR-BI and BII, respectively; TNF-α: 3.4- and 2.4-fold higher in SR-BI and BII, respectively; Figure 3A). Additionally, the qPCR assay of inflammatory genes revealed that the expression levels of CXCL1, IL-6, and TNF-α in the liver were significantly increased in both SR-BI and SR-BII transgenic mice at 24 h after CLP. The increase in the expression levels was larger than in WT (CXCL1: 5.7- and 8.0-fold higher in SR-BI and SR-BII, respectively; IL-6: 5.3- and 4.0-fold higher in SR-BI and SR-BII, respectively; TNF-α: 3.3- and 2.6-fold higher in SR-BI and SR-BII, respectively; Figure 3B). In contrast, renal CXCL1, IL-6, and TNF-α mRNA levels were comparably elevated among all experimental groups after CLP surgery, although the CXCL1 level was more increased in SR-BII transgenic mice compared to WT mice (Figure 3C). Next, we hypothesized that the hyperinflammatory status of SR-B transgenic mice during sepsis could impair vascular endothelial integrity and increase vascular permeability. A marker of systemic vascular endothelial dysfunction, VEGF, was assessed. The plasma VEGF level was elevated at 24 h after CLP. The level was significantly higher in SR-B transgenic mice than in WT (61% and 49% higher in SR-BI and SR-BII, respectively; Figure 3D). The naphthol AS-D chloroacetate esterase staining showed abundant liver neutrophil infiltration at 24 h after CLP. Liver from SR-BI transgenic mice had the highest infiltration of neutrophils among the experimental groups (Figure 3E,F). However, neutrophil infiltration was minimal in the kidney across all the experimental groups even after CLP (Supplemental Figure S1).

Therefore, the overexpression of human SR-B transgenes exacerbated the inflammatory response, especially in the liver, and increased a marker of vascular endothelial dysfunction during CLP sepsis. Neutrophil infiltration was most pronounced in the liver of SR-BI transgenic mice.

### 3.4. Human SR-B Significantly Reduced Bacteria Accumulation in the Liver via Promoting Macrophage Phagocytosis of Bacteria; The Phagocytosis Was Prominent in SR-BII Transgenic Mice

In our previous in vitro study using HeLa cells overexpressing each human SR-B splice variant, we found that both SR-BI and SR-BII could act as pattern recognition receptors that promoted bacterial adhesion, internalization, cytosolic invasion, and intracellular survival and proliferation [12]. Therefore, we hypothesized that SR-BI and SR-BII transgenic mice would have increased bacterial invasion in distant organs in CLP sepsis.

**Figure 4:**
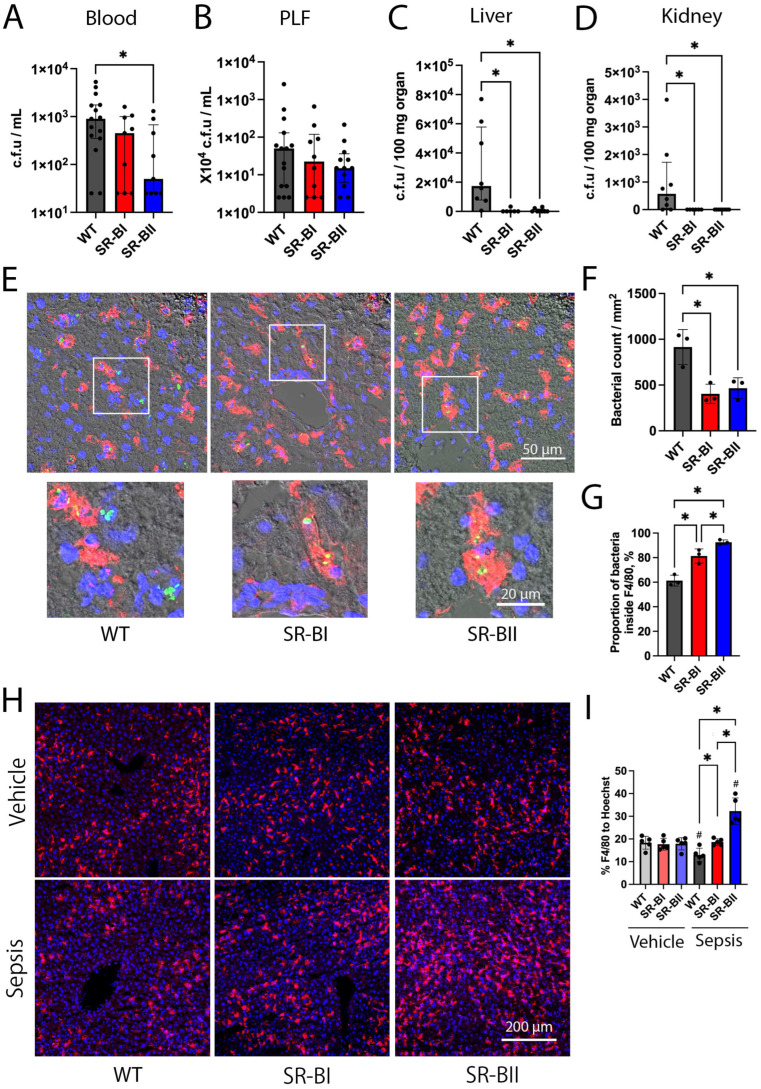
Bacterial counts after cecal ligation and puncture, and green fluorescent protein-labeled *E. coli* engulfed by macrophages after bacterial injection. The bacterial counts in the following specimens were examined and compared between wild-type (WT) and human class B scavenger receptor BI (SR-BI) and BII (SR-BII) transgenic mice at 24 h following cecal ligation and puncture (CLP); (**A**) Blood (WT, *n* = 15; SR-BI, *n* = 9; SR-BII, *n* = 9; *n* = 33 total). (**B**) Peritoneal lavage fluid (PLF) (WT, *n* = 15; SR-BI, *n* = 10; SR-BII, *n* = 12; *n* = 37 total). (**C**) Liver and (**D**) kidney (WT, *n* = 8; SR-BI, *n* = 6; SR-BII, *n* = 8; *n* = 22 total). The data are shown as medians (interquartile ranges). * *p* < 0.05 between WT, SR-BI, and SR-BII. The immunofluorescence analyses were performed to observe localization of green fluorescent protein (GFP)-labeled *E. coli* and macrophages in liver harvested at 8 h after intraperitoneal bacterial infusion. (**E**) Representative images for liver specimens are shown. Positive staining for *E. coli*, F4/80, and DNA are indicated in green, red, and blue, respectively. The GFP-labeled bacteria were frequently localized in F4/80-positive cells in liver of SR-BI and SR-BII transgenic mice. White boxes in the top row represent the boundaries of the magnified insets in the bottom row Bar = 50 µm, insets: bar = 20 µm. (**F**) The total number of bacteria per mm^2^ and (**G**) the percentage of bacteria localized within macrophages were averaged and compared across the experimental groups (WT, *n* = 3; SR-BI, *n* = 3; SR-BII, *n* = 3; *n* = 9 total). The data are represented as means ± SD. * *p* < 0.05 between WT, SR-BI, and SR-BII. Hepatic macrophages of wild-type (WT) and human class B scavenger receptor BI (SR-BI) and BII (SR-BII) transgenic mice were observed under a confocal microscope at 8 h after intraperitoneal injection with vehicle or *E. coli* K12. (**H**) Representative images are shown. Positive staining for F4/80 and DNA is indicated in red and blue, respectively. Bar = 200 µm. (**I**) The percentage of F4/80-positive cells relative to the total number of nuclei was averaged for each mouse and compared across experimental groups (*n* = 5 per group; *n* = 30 total). The data are shown as means ± SD. # *p* < 0.05 versus vehicle, * *p* < 0.05 between WT, SR-BI, and SR-BII after bacterial or vehicle infusion.

Unexpectedly, we observed a decreasing trend in bacterial counts in the blood and peritoneal lavage fluid of septic SR-B transgenic mice at 24 h after CLP. This tendency was more pronounced in SR-BII transgenic mice, which had a 96% lower blood bacterial count compared to WT mice (Figure 4A,B). Of note, bacteria were nearly undetectable in the liver and kidney of SR-BI and SR-BII transgenic mice during CLP-induced sepsis, despite abundant bacterial colonies detected in those organs of WT mice (Figure 4C,D).

Next, we investigated the mechanism by which invading bacteria could be eliminated in the liver and kidney. GFP-labeled *E. coli* were intraperitoneally injected to mimic enteric bacteria released into the peritoneal cavity. We previously detected higher levels of mRNA expression and protein production of human SR-BI and SR-BII in bone marrow-derived macrophages (BMDMs) from SR-BI and SR-BII transgenic mice, compared to WT mice [17]. Resident and monocyte-derived macrophages were visualized with an immunofluorescence assay using anti-F4/80 antibody. We found an increased number of GFP-expressing bacteria exclusively localized within F4/80-positive cells of liver tissue harvested at 8 h after injection (Figure 4E). The total number of bacteria was 56% and 49% lower in the liver of SR-BI and SR-BII transgenic mice, respectively, compared to that in the liver of WT mice, consistent with the liver bacterial counts after CLP surgery (Figure 4F). Furthermore, the proportion of bacteria located within F4/80-positive cells relative to the total number of identified bacteria was 33% and 51% higher in SR-BI and SR-BII transgenic mice, respectively, compared to WT mice. Bacteria were more frequently detected within macrophages in SR-BII compared to SR-BI transgenic mice (Figure 4G). The kidney sections of all the experimental groups had too few GFP-labeled bacteria to perform analyses (Supplemental Figure S2).

Moreover, F4/80-positive cells were significantly decreased in the liver of WT mice at 8 h after *E. coli* injection (29% decrease from the baseline), whereas their number was unchanged in SR-BI transgenic mice and markedly increased (80% increase from the baseline) in SR-BII transgenic mice (Figure 4H,I). This result indicates that extensive recruitment of monocyte-derived macrophages occurs in the liver of SR-BII transgenic mice.

According to several reports, SR-BI is abundantly expressed in hepatocytes and kidney epithelial cells, suggesting that those cells might internalize bacteria via the scavenger receptors [23,24]. Immunohistochemical analysis for GFP-labeled bacteria revealed that those bacteria were frequently located in sinusoids between hepatocytes, not within hepatocytes, at 8 h after injection (Supplemental Figure S3A). A few bacteria were observed in peritubular capillaries of kidneys isolated from all the experimental groups (Supplemental Figure S3B).

In summary, the results suggest that overexpression of human SR-BI and SR-BII dramatically decreased bacteria accumulation in the liver due to increased phagocytosis and the number of macrophages compared to WT mice. This effect was larger in SR-BII transgenic mice.

### 3.5. Overexpression of Human SR-BI Reduced Systemic HDL-C Levels and Storage of Lipid Droplets in the Adrenal Gland, and Dampened the Increase of Circulating Corticosterone in Response to CLP Sepsis

Both SR-BI and SR-BII can mediate HDL-C uptake [5,10]. We previously reported that human SR-BI and SR-BII transgenic mice exhibited significantly lower levels of total serum cholesterol compared to WT mice under physiological conditions. Both transgenic mouse lines had similarly elevated adrenal glucocorticoid levels after LPS injection; however, this increase was less than in WT mice [17].

**Figure 5:**
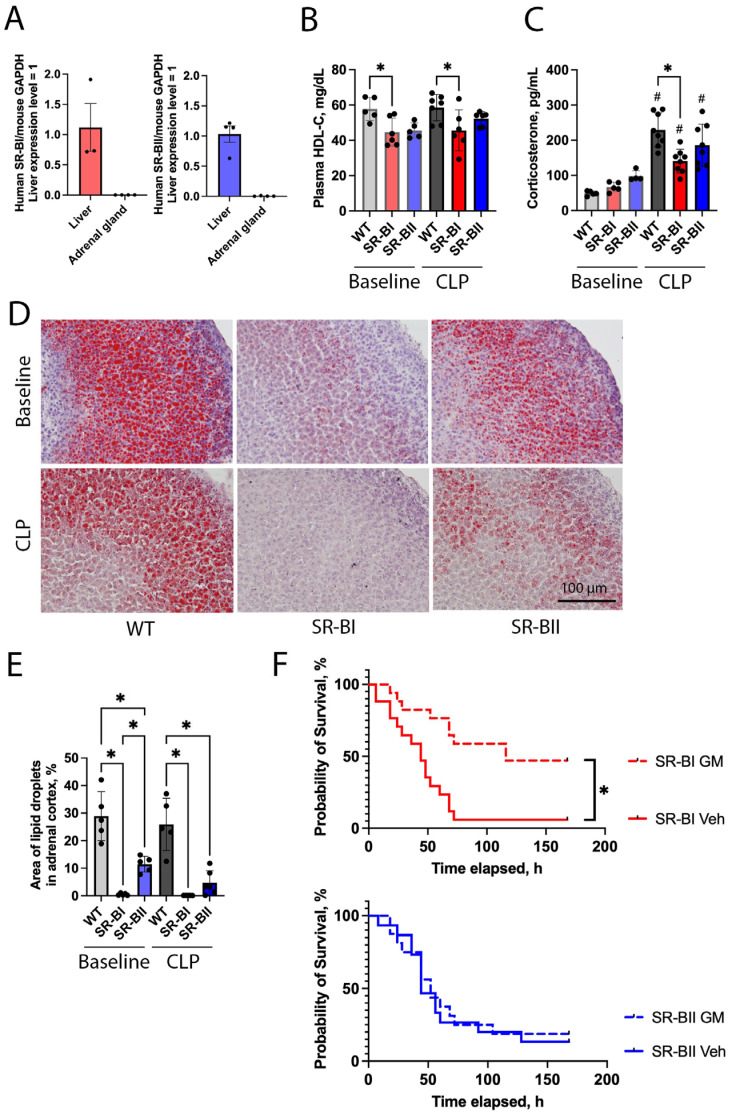
Systemic high-density lipoprotein cholesterol and corticosterone levels and lipid droplets in adrenal cortex at baseline and in sepsis. (**A**) The expression of human class B scavenger receptor BI (SR-BI) and BII (SR-BII) transgenes were evaluated in adrenal glands of SR-BI and SR-BII transgenic mice relative to corresponding hepatic expression levels (SR-BI liver, *n* = 3; SR-BI adrenal gland, *n* = 4; SR-BII liver, *n* = 4; SR-BII adrenal gland, *n* = 4). The expression levels were normalized with mouse glyceraldehyde-3-phosphate dehydrogenase (GAPDH). The data are shown as means ± SD. (**B**) High-density lipoprotein cholesterol (HDL-C) in the plasma of wild-type (WT) and human SR-BI and SR-BII transgenic mice were assessed at baseline or at 24 h after cecal ligation and puncture (CLP) surgery and compared among the experimental groups (baseline WT, *n* = 5; baseline SR-BI, *n* = 6; baseline SR-BII, *n* = 5; CLP WT, *n* = 7; CLP SR-BI, *n* = 6; CLP SR-BII, *n* = 6; *n* = 35 total). The data are shown as means ± SD. * *p* < 0.05 between WT, SR-BI and SR-BII after CLP or at baseline. (**C**) Serum corticosterone levels were also evaluated at baseline or in CLP-induced sepsis (baseline WT, *n* = 5; baseline SR-BI, *n* = 5; baseline SR-BII, *n* = 4; CLP WT, SR-BI, and SR-BII, *n* = 8 per group; *n* = 38 total). The data are shown as means ± SD. # *p* < 0.05 versus sham. * *p* < 0.05 between WT, SR-BI and SR-BII after CLP or at baseline. Lipid droplets in the adrenal cortex were examined using Oil Red O staining (and hematoxylin counterstaining) for the adrenal glands of WT, SR-BI, and SR-BII transgenic mice at baseline or at 24 h after CLP. (**D**) Representative images for all the experimental groups are shown. The red area represents lipid droplets. Bar = 100 µm. (**E**) The percentage of lipid droplet area in the adrenal cortex was calculated using Fiji and compared across the groups (baseline WT, SR-BI, SR-BII, *n* = 5 per group; CLP WT, SR-BI, and SR-BII, *n* = 5 per group; *n* = 30 total). The data are shown as means ± SD. * *p* < 0.05 between WT, SR-BI and SR-BII after CLP or at baseline. (**F**) A seven-day survival study was conducted for vehicle (Veh) and glucocorticoid and mineralocorticoid (GM) treatments in human class B scavenger receptor BI (SR-BI) and BII (SR-BII) transgenic mice following cecal ligation and puncture (CLP) surgery. Kaplan–Meier survival curves are shown. GM treatment significantly improved the survival rate of septic SR-BI (GM treatment (dotted red line) vs. vehicle treatment (solid red line), 47.1% vs. 5.9%, *p* < 0.001; *n* = 17 per group; *n* = 34 total), whereas it did not improve the survival in SR-BII transgenic mice (GM treatment (dotted blue line) vs. vehicle treatment (solid blue line), 18.6% vs. 13.3%, *p* = 0.81; *n* = 16 in the GM and *n* = 15 in the vehicle groups; *n* = 31 total). * *p* < 0.05 versus respective control.

We evaluated the expression levels of human SR-BI and human SR-BII in the adrenal gland of the respective transgenic mice. Transgene expression in the adrenal gland was negligible compared with that in the liver (Figure 5A). Next, we assessed the effect of human SR-BI or SR-BII overexpression on HDL-C and corticosterone levels following CLP. At baseline and 24 h after CLP, plasma HDL-C levels were significantly lower in SR-BI transgenic mice than in WT mice (22% lower at both baseline and after CLP), probably because of increased HDL-C uptake in organs overexpressing SR-BI, such as the liver. HDL-C levels in SR-BII transgenic mice were not as low as those in SR-BI transgenic mice (Figure 5B). Circulating corticosterone was increased in all experimental groups after CLP. However, the corticosterone levels in septic SR-BI transgenic mice were significantly lower than those in WT (39% reduction). The difference in the corticosterone levels between SR-BII and WT mice did not reach statistical significance (Figure 5C).

Cholesterol ester stored in adrenal gland lipid droplets provides an available pool of free cholesterol for corticosterone synthesis (functional reserve) [25]. We measured the area of lipid droplets in adrenal glands at baseline and following CLP surgery as a proxy for this functional reserve. At baseline, both SR-BI and SR-BII transgenic mice had significantly fewer lipid droplets in the adrenal cortex than WT mice (99% and 60% lower in SR-BI and SR-BII, respectively), with lipid storage being particularly low in SR-BI transgenic mice. After CLP, lipid area remained stable in WT, decreased in SR-BII mice, and declined to nearly zero in SR-BI mice (Figure 5D,E). Moreover, we assessed lipid droplets in the liver, where SR-BI and SR-BII transgenes are overexpressed. Unexpectedly, lipid droplets were sparse across all the experimental groups (Supplemental Figure S4).

To test whether the partial adrenal insufficiency contributed to higher mortality in septic SR-BI mice relative to WT mice, we conducted a 7-day survival study comparing CLP with vehicle vs. CLP with glucocorticoid plus mineralocorticoid (GM) supplementation in SR-BI and SR-BII transgenic mice. The GM treatment improved survival in SR-BI transgenic mice (GM treatment vs. vehicle treatment, 47.1% vs. 5.9%, *p* < 0.001), but not in SR-BII transgenic mice (GM treatment vs. vehicle treatment, 18.6% vs. 13.3%, *p* = 0.81) (Figure 5F).

Overexpressed human SR-BI remarkably reduced systemic HDL-C levels and storage of lipid droplets in the adrenal gland, and dampened systemic increase of corticosterone in response to septic insult, while human SR-BII transgenic mice showed milder changes in these parameters. The increased mortality of SR-BI transgenic mice during CLP sepsis was mainly attributable to the partial adrenal insufficiency.

## 4. Discussion

The dysregulated host immune response during sepsis causes poor clinical outcomes in septic patients [1]. Class B scavenger receptors BI and BII are involved in innate immunity, lipid metabolism, and hormonal responses [5,10,11]. When studied in vitro, human SR-BI and SR-BII can act as pattern-recognition receptors to mediate uptake of gram-negative bacteria into cells [11,12,14]. To examine the role of those receptors in animal sepsis models, global SR-BI/SR-BII knock-out (KO) mice were employed [13,26], although these studies were difficult to interpret because of multiple interacting abnormalities such as excessive lymphocyte apoptosis, adrenal insufficiency (inhibition of HDL-C uptake, impaired accumulation of cholesterol esters), and female infertility [27,28]. For example, survival depended strongly on specific regimens of adrenal replacement. Furthermore, the SR-BI/SR-BII KO mice cannot differentiate between SR-BI- and SR-BII-specific functions. Therefore, we developed human SR-B transgenic mice, with the transgenes expressed primarily in the liver, as well as the kidney and BMDMs, and we hoped this would help unravel their role in sepsis. We found the following: (1) Human SR-BI or SR-BII overexpression led to similar poor survival, liver damage, enhanced inflammatory responses, and decreased bacterial accumulation after CLP surgery compared to WT mice. (2) However, the mechanisms were different. SR-BII transgenic mice had higher bacterial phagocytic activity and a greater abundance of hepatic macrophages. In contrast, SR-BI transgenic mice had enhanced hepatic neutrophil infiltration. (3) Human SR-BI transgenic mice had partial adrenal insufficiency following sepsis; supplementation with glucocorticoid and mineralocorticoid reversed the survival deficit of septic SR-BI (but not SR-BII) transgenic mice. These results are discussed below.

### 4.1. Similarity in Survival Deficit and Liver Damage, Inflammatory Markers, and Bacterial Accumulation

The survival rate of SR-BI or SR-BII transgenic mice after CLP was worse compared to that of the WT control mice. This finding is consistent with our earlier in vitro study, suggesting that SR-B receptors may worsen sepsis by facilitating bacterial cytosolic accumulation, evasion of lysosomal complex, and proliferation in host cells [12]. At the same time, the survival benefit of SR-B receptors in sepsis has been also reported using SR-BI/SR-BII KO mice that showed lower survival rates after CLP than WT controls. Indeed, the result was remarkably impacted by adrenal insufficiency, likely due to impaired cholesterol ester uptake in the adrenal glands [13]. Corticosterone pretreatment (glucocorticoid only) could not reverse the survival outcome following CLP [13]. However, we reported SR-BI/BII KO mice had a higher survival rate than WT in CLP-induced septic mice pretreated with both dexamethasone and fludrocortisone acetate [14]. As such, the survival of SR-BI/SR-BII KO mice after CLP varied according to the details of adrenal replacement (none: worse; glucocorticoids only: worse; both gluco- and mineralo-corticoids: improved) [13,14,22]. Hence, we aimed to elucidate the survival impact of SR-B receptors in CLP sepsis using SR-BI or SR-BII-overexpressing mice to minimize confounding adrenal insufficiency. Our result suggests SR-BI or SR-BII overexpression may be associated with adverse survival outcomes in sepsis.

SR-BI or SR-BII transgenic mice had more severe histological liver damage along with higher levels of liver injury markers at 24 h after CLP. Moreover, systemic inflammatory cytokines and vascular endothelial dysfunction markers are increased in SR-BI or SR-BII transgenic mice vs. wild-type septic mice. Notably, the expression of inflammatory genes was higher in the livers of SR-BI or SR-BII transgenic mice, whereas the expression in kidneys was similar across the experimental groups. A previous in vitro study exhibited a more prominent dose-dependent stimulation of cytokine production in SR-B-overexpressing HEK293 cells than in mock-transfected cells across increasing doses of ligands, including *E. coli*, LPS, and GroEL [11]. Additionally, SR-BI/BII KO significantly attenuated systemic proinflammatory responses in mice supplemented with both glucocorticoid and mineralocorticoid prior to CLP surgery. Such evidence is consistent with a profound inflammatory response and vascular endothelial impairment in septic mice overexpressing SR-BI or SR-BII. Our results suggest that a liver hyperinflammatory state and severe liver injury may be mainly involved in poor outcomes of SR-BI or SR-BII transgenic mice. Sepsis is a systemic syndrome involving multiple organs [29]. While the obtained data indicate the liver as a major site of dysregulated responses, extrahepatic mechanisms may also contribute to sepsis outcomes.

As we recently reported, we detected an abundance of bacteria in the liver of WT mice after CLP [30]. Unexpectedly, the viable bacterial count was dramatically reduced in the liver of SR-BI and SR-BII transgenic mice. The immunofluorescence analyses using GFP-labeled *E. coli* demonstrated a significant increase in the frequency of bacteria localized within hepatic macrophages in SR-BI or SR-BII transgenic mice, accompanied by a reduction in the total liver bacterial number. Further studies incorporating assays of lysosomal localization are warranted to investigate the fate of internalized bacteria.

### 4.2. Underlying Cellular Responses Differ in SR-B1 and SR-BII Transgenic Mice

Despite similar suppression of liver bacteria counts in SR-BI and SR-BII transgenic mice, the mechanisms seem to be quite different. SR-BII transgenic mice had a higher phagocytic efficiency of hepatic macrophages compared to SR-BI transgenic mice, consistent with our previous report showing a greater number of internalized bacteria in HeLa cells overexpressing SR-BII versus SR-BI [12]. That same study also demonstrated that bacteria internalized via SR-BI or SR-BII could survive and proliferate within host cells [12]. The discrepancy between these findings and our present results may be attributed to differences in the cell types involved in bacterial uptake.

SR-BII transgenic mice also demonstrated the greatest increase in the number of F4/80-positive cells in the liver following bacterial infusion. Once hepatic macrophages internalize bacteria they may recruit circulating monocytes to the liver. Hepatic macrophages were reduced in the WT mice, possibly due to bacteria-induced Kupffer cell PANoptosis [31].

Conversely, SR-BI transgenic mice had greater neutrophil infiltration in the liver during CLP sepsis vs. SR-BII mice. The pronounced neutrophil influx may be caused by partial adrenal insufficiency occurring in SR-BI (see below). Reportedly, adrenalectomy in rats facilitated the maturation of bone marrow neutrophils. Furthermore, a deficiency of adrenal cortical hormones reduced L-selectin expression on polymorphonuclear (PMN) cells in the bone marrow while increasing its expression on PMN cells in systemic circulation, thereby promoting their migration into peripheral blood and subsequently to the extracellular matrix [32]. However, the observed increase in neutrophil infiltration cannot distinguish between enhanced host defense and dysregulated inflammatory responses. The detailed characterization of neutrophil activation and function will be an important focus of future studies.

The reduction in bacterial load was also observed in the kidney of SR-B transgenic mice, despite the lack of impact of SR-B transgenes on the extent of sepsis-induced kidney damage: elevation of BUN, Cystatin C, and inflammatory cytokine expression did not differ across the experimental groups. Bacteria invading from the peritoneal cavity first get trapped in the liver because of its proximity and anatomical connection. Then, the remaining few bacteria are able to reach the kidneys via systemic circulation. The observed bacterial load by immunofluorescence analysis was apparently not sufficient to impact the kidneys structurally or functionally. Bacteria localized within F4/80-positive cells were not detected in the kidneys of any experimental group. Thus, bacterial count in the kidney might have reflected the phagocytic activity in the liver. Longitudinal measurement of bacterial load may help determine the relationship between organ-specific bacterial loads. Moreover, bacterial phagocytosis by macrophages may have had a limited influence on cytokine production and kidney damage during CLP-induced sepsis compared to other mechanisms, such as microcirculatory dysfunction [33,34].

### 4.3. Difference in Cholesterol Metabolism and Adrenal Function

SR-Bs expressed on the liver and steroidogenic organs are involved in selective uptake of HDL-C [5]. SR-BI has a higher HDL-C uptake than SR-BII because SR-BI is more highly expressed on the cell surface [17]. In our study, the plasma level of HDL-C in SR-BI transgenic mice was significantly lower than that of WT mice at baseline and in sepsis. Additionally, we observed few lipid droplets in the livers of SR-BI transgenic mice, similar to those in WT and SR-BII transgenic mice. These results may corroborate that hepatic overexpression of SR-BI facilitated cholesterol uptake and the excretion of cholesterol from the liver into bile, reducing the cholesterol level in plasma, as in an earlier report [35]. In a dyslipidemia patient cohort, SR-BI protein levels were inversely associated with HDL-C levels [36]. Hence, the activity of SR-BI in the liver may be crucial to maintain plasma HDL-C levels. The Oil Red O staining demonstrated a near absence of lipid droplets in the adrenal cortex of SR-BI transgenic mice at baseline. Human SR-BI and SR-BII expression was barely detectable in the adrenal gland of the respective transgenic mice, which is consistent with a prior report showing minimal adrenal expression of transgene driven by pLiv-11 [37]. Therefore, the diminished adrenal cholesterol ester storage in SR-BI transgenic mice likely reflects a decreased uptake of HDL due to its low plasma level. This is similar to what is observed in SR-BI/II KO mice [38] where diminished adrenal cholesterol ester storage is due to the absence of SR-BI/II, which prevents cholesterol uptake despite high plasma HDL levels. Although a CLP-induced septic insult increased systemic corticosterone levels in all of the experimental groups, the hormone levels in SR-BI transgenic mice were significantly lower than in WT mice, suggesting that the production of corticosterone was reduced because of the cholesterol ester shortage. In contrast, SR-BII transgenic mice exhibited only a mild reduction in corticosterone levels, probably because some residual cholesterol ester remained in the adrenal cortex before CLP. Furthermore, replacement with both gluco- and mineralocorticoids (GM) remarkably improved the survival of SR-BI transgenic mice to a level comparable to that of WT mice, whereas it had no effect on SR-BII mice. This finding demonstrates that the partial adrenal insufficiency mainly contributed to the increased mortality in SR-BI mice. However, the downstream effects of GM treatment on inflammation, liver injury, neutrophil infiltration, or bacterial burden in SR-BI transgenic mice remain speculative and require dedicated follow-up studies. Not only genetic variants but also body mass index and dietary fat reportedly affect SR-BI expression [36,39]. Hyperglycemia and estrogen are reported to facilitate a splicing-mediated shift from SR-BI to SR-BII [40,41]. Thus, relative SR-BI and SR-BII expression could be heterogeneous among human patients. This may explain the reason why the results of systemic steroid supplementation in human sepsis has led to conflicting results and much discussion [42]. Perhaps some of the differential responses to steroid supplementation are driven by genetic differences in the population, including SR-BI and SR-BII variants. A recent meta-analysis of prospective observational studies pointed out that low systemic levels of cholesterol, including HDL-C, upon intensive care unit admission was associated with hyperinflammation, higher severity, and worse prognosis of critically ill patients with sepsis [43]. Further research is needed to elucidate the interplay between cholesterol metabolism, the endocrine system, and the immune system via SR-Bs.

Why was the liver more susceptible to injury than the kidney in the context of increased bacterial clearance? The liver is a major reticuloendothelial organ and a key site of bacterial sequestration during abdominal sepsis [44]. Our previous study showed that the liver exhibits the highest bacterial burden among organs in CLP-induced sepsis [30]. In our current study, we also observed a greater number of neutrophils (Figure 3F and Supplemental Figure S1) and F4/80-positive macrophages (Figure 4E and Supplemental Figure S2) in the liver compared to the kidney at 24h after CLP, supporting the presence of stronger local innate immune activation in the liver. Based on our previous in vitro study, SR-BI-overexpressing BMDMs showed increased phagocytic activity without a corresponding increase in cytokine production compared to WT mice [17]. In addition, these mice developed an adrenal insufficiency, which has been reported to augment inflammatory responses of neutrophils and macrophages [32,45]. Thus, increased bacterial uptake and heightened inflammatory responsiveness may act in parallel mainly in the liver of septic SR-BI transgenic mice. In contrast, SR-BII-overexpressing macrophages have been shown to have both enhanced phagocytosis and increased inflammatory cytokine production [17]. It is therefore plausible that increased bacterial uptake in these mice is accompanied by excessive inflammatory responses, contributing to liver injury.

### 4.4. Limitations

We acknowledge several limitations of our study. Firstly, the SR-BI and SR-BII transgenic mice had human SR-B transgenes expressed not only in the liver but also in the kidney, lung, and spleen, even though the promoter was designed to express those receptors specifically in the liver [17,46]. The transgenes were also highly expressed in BMDMs, which can be widely distributed throughout tissues [17]. Therefore, our study could not test organ-specific human SR-B functions. Second, we used only male mice because the estrous cycle may affect hepatic SR-B levels; several previous reports found that estrogen can regulate the expression level of SR-BI and SR-BII in the liver [47,48]. However, this limits the generalizability of this study. Third, neutrophil accumulation in the liver was assessed histologically in this study. Future studies using flow cytometry would allow a more quantitative analysis and characterization of neutrophil maturation and activation states. Fourth, the CLP mouse model mimics primarily the early hyperinflammatory phase of sepsis, where mouse mortality occurs rapidly following the surgically induced insult. In contrast, mortality in human sepsis commonly arises during a later, immunosuppressive phase that is associated with secondary infections [49]. Therefore, the translational relevance of our findings may be limited to the early hyperinflammatory stage, and whether our findings apply to the later stage of human sepsis would be better tested in a two-hit model of sepsis [50]. Finally, a causal relationship between bacterial burden, immune activation, and organ injury could not be definitively established in this study. Additional research is warranted to clarify the hierarchy and potential interrelationship.

## 5. Conclusions

Our findings suggest human SR-BI and SR-BII overexpression contributes to higher mortality in CLP sepsis mainly by excessive organ damage and inflammation in the liver despite increased hepatic bacterial clearance. SR-BI transgenic mice exhibited marked neutrophil infiltration in the liver and adrenal insufficiency, whereas SR-BII had prominent bacterial phagocytosis in hepatic macrophages without significant adrenal insufficiency. Thus, the increased death from sepsis may be attributable to different mechanisms in the two splice variants (Table 1). A comprehensive understanding of the pleiotropic functions of human SR-B may aid in the development of targeted therapies with reduced off-target effects. Further investigation is necessary to develop therapeutic strategies that could selectively modulate specific functions of these receptors.

## Figures and Tables

**Table 1:** Possible contributors to worsened survival in cecal ligation and puncture-induced sepsis.

Genotype	Liver			Adrenal Insufficiency
	Macrophages	Neutrophils	Inflammatory Cytokines	
SR-BI		✓	✓	partial
SR-BII	✓		✓	

SR-BI, class B scavenger BI; SR-BII, class B scavenger receptor BII.

## Data Availability

The datasets generated during the current study are available in the Mendeley Data repository [https://data.mendeley.com/preview/dn35n7phbt?a=b55177cc-618a-489b-aa17-89339ca9be19] (accessed on 8 September 2025).

## Supplementary Materials

The following supporting information can be downloaded at: https://www.mdpi.com/article/10.3390/biom16050670/s1, Figure S1: Neutrophil infiltration in the kidneys at 24 h after cecal ligation and puncture. Figure S2: Renal macrophages and bacteria at 8 h after bacterial injection. Figure S3: Immunohistochemistry of green fluorescent protein-labeled *E. coli* in the liver and kidney at 8 h after intraperitoneal infusion. Figure S4: Lipid droplets in the liver at baseline.

## Abbreviations

The following abbreviations are used in this manuscript:

SR-BI	Scavenger receptor BI
SR-BII	Scavenger receptor BII
CLP	Cecal ligation and puncture
PRR	Pattern-recognition receptor
PAMP	Pathogen-Associated Molecular Pattern
LPS	Lipopolysaccharide
KO	Knockout
WT	wild-type
PCR	Polymerase chain reaction
RT-PCR	Reverse transcription polymerase chain reaction
CXCL1	Chemokine (C-X-F motif) ligand 1
TNF-α	Tumor necrosis factor-α
IL-6	Interleukin-6
GAPDH	Glyceraldehyde-3-phosphate dehydrogenase
CT	Cycle threshold
GFP	Green fluorescent protein
PBS	Phosphate-buffered saline
PAS	Periodic acid-Schiff
BUN	Blood urea nitrogen
AST	Aspartate aminotransferase
ALT	Alanine aminotransferase
VEGF	Vascular endothelial growth factor
GM	Glucocorticoid and mineralocorticoid
ANOVA	Analysis of variance
MAPK	Mitogen-activated protein kinase
BMDM	Bone marrow-derived macrophages
PLF	Peritoneal lavage fluid
