# Supplementary material for: Different Functions of Human Scavenger Receptors BI and BII Overexpressed in a Murine Abdominal Sepsis Model
Source: Biomolecules. 2026 May 1;16(5):670. doi: 10.3390/biom16050670 (PMC13204255; doi:10.3390/biom16050670)
Supplement: Supplementary file 1 [file biomolecules-16-00670-s001.zip › biomolecules-4229855-supplementary.pdf]

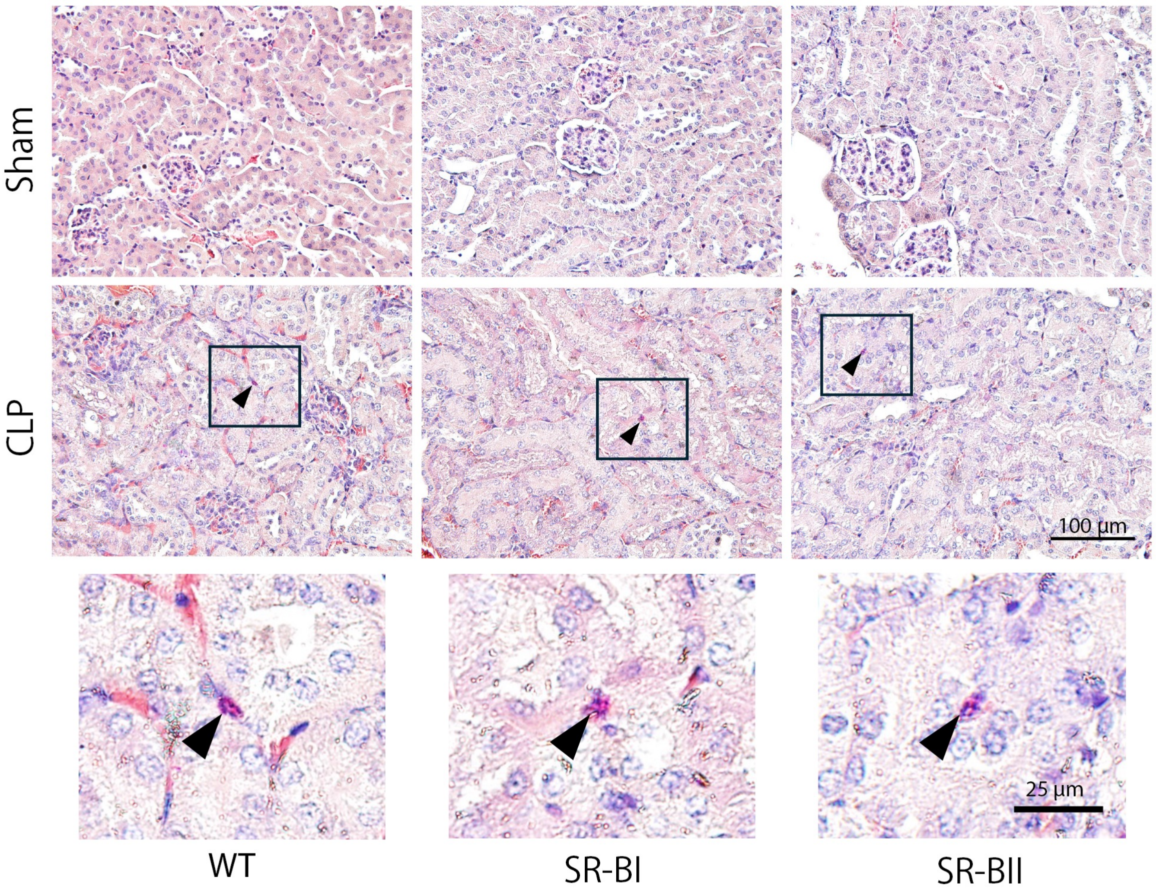

Supplemental Figure S1. Neutrophil infiltration in the kidneys at 24 h after cecal ligation and puncture.
The kidneys were collected from Wild-type (WT) and human class B scavenger receptor BI (SR-BI) and BII (SR-BII) transgenic mice at 24 h after cecal ligation and puncture (CLP) surgery. Representative images of naphthol AS-D chloroacetate esterase staining in the kidneys are exhibited (sham WT and SR-BI, n = 5 per group; sham SR-BII, n = 4; CLP WT, n = 5; CLP SR-BI, n = 6; CLP SR-BII, n = 5; n = 30 total). The arrowheads denote infiltrating neutrophils. Bar = 100 µm, insets: bar = 25 µm.

Supplemental figure 1

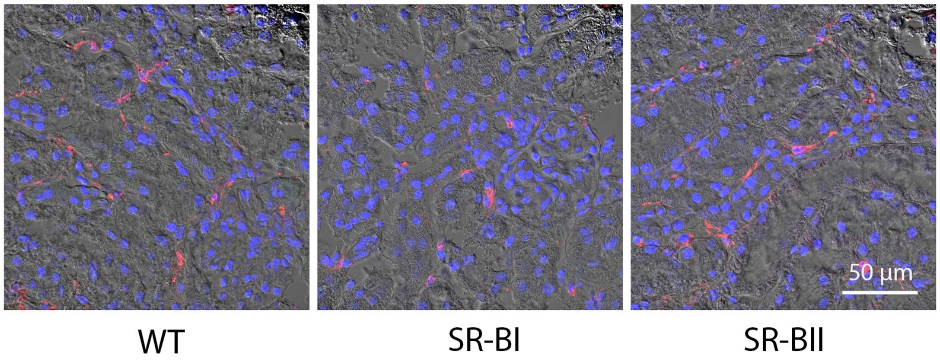

Supplemental Figure S2. Renal macrophages and bacteria at 8 h after bacterial injection.
The green fluorescent protein (GFP)-labeled E. coli and macrophages were assessed with immunofluorescence analyses in kidneys harvested at 8 h after intraperitoneal bacterial infusion. Macrophages were labeled with rabbit anti-mouse F4/80 antibody followed by Alexa 647–goat anti-rabbit IgG (red). The nuclear counterstain was Hoechst 33342 (blue). Representative images for kidney specimens are exhibited (WT, n = 3; SR-BI, n = 3; SRBII, n = 3; n = 9 total). Bar = 50 µm.

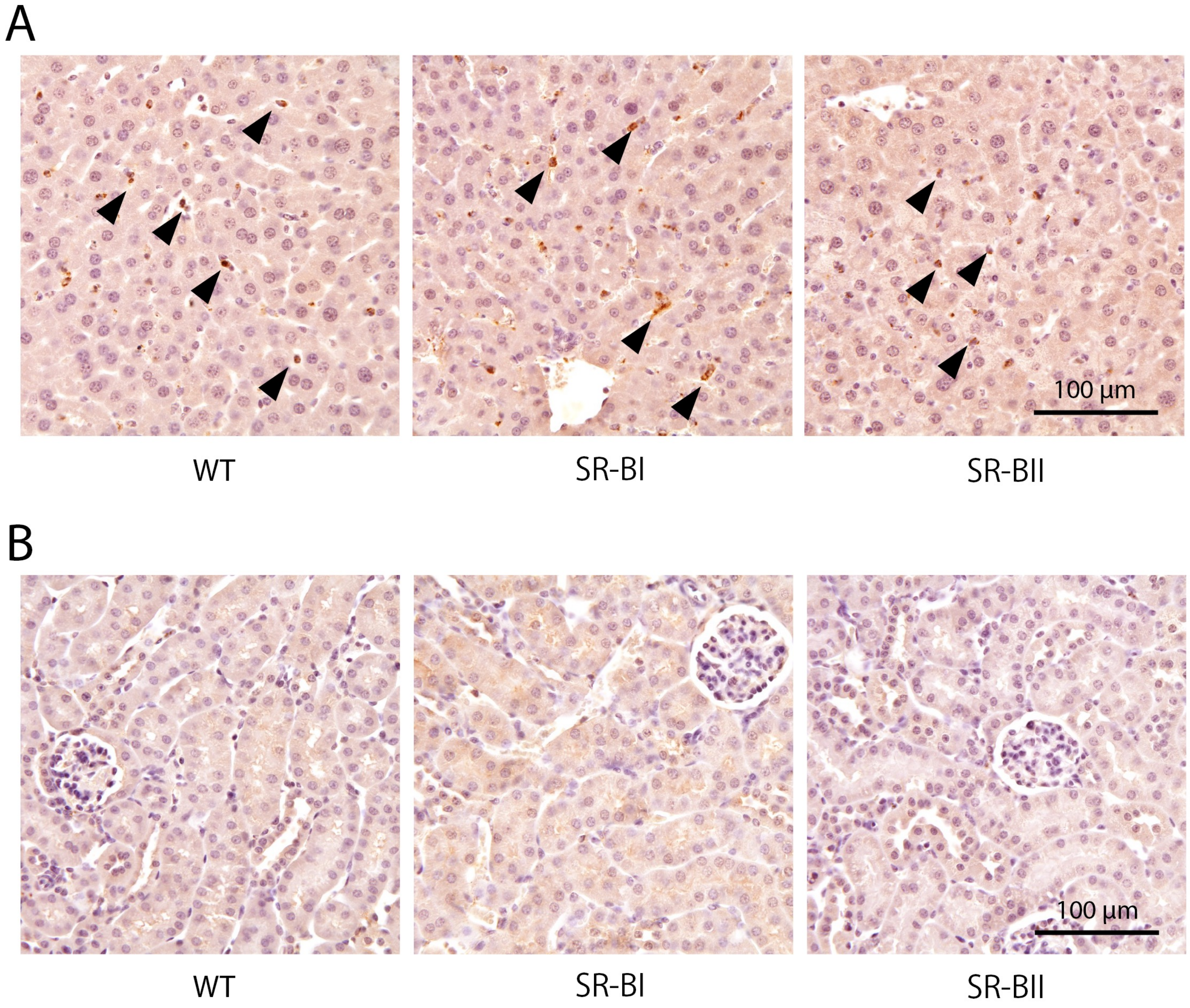

Supplemental Figure S3. Immunohistochemistry of green fluorescent protein-labeled E. coli in the liver and kidney at 8 h after intraperitoneal infusion.
The immunohistochemical examination of green fluorescent protein (GFP)-labeled E. coli was performed in the liver and kidney collected from Wild-type (WT) and human class B scavenger receptor BI (SR-BI) and BII (SR-BII) transgenic mice at 8 h after injection with the bacteria. Representative images of (A) liver and (B) kidney from the experimental groups are shown (WT, n = 3; SR-BI, n = 3; SR-BII, n = 3; n = 9 total). Positive area (arrow heads) represents GFP-labeled E. coli. The bacteria were frequently detected in liver sinusoids. Bar = 100 μm.

Supplemental figure 3

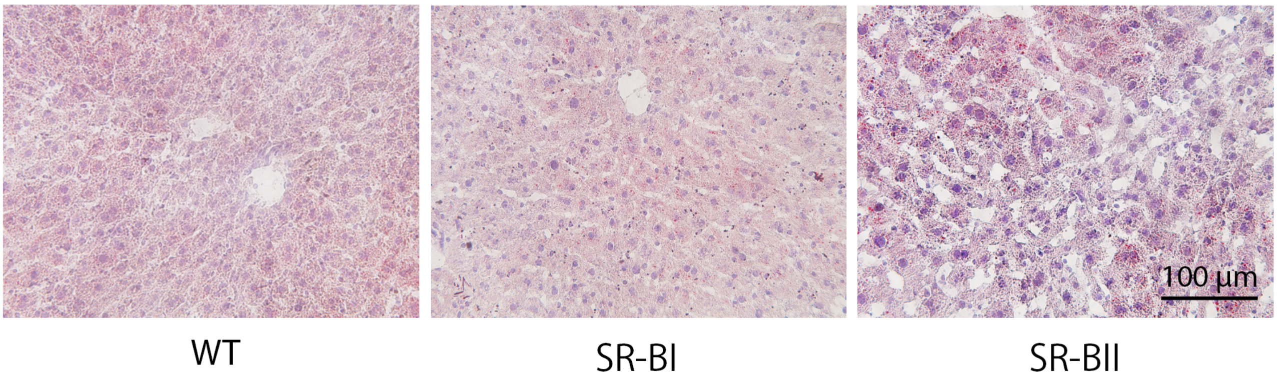

Supplemental Figure S4. Lipid droplets in the liver at baseline.
The liver was collected from Wild-type (WT) and human class B scavenger receptor BI (SR-BI) and BII (SR-BII) transgenic mice at baseline. Representative images of Oil Red O staining of liver sections are shown (WT, n = 3; SR-BI, n = 3; SRBII, n = 3; n = 9 total). The red area represents lipid droplets. Bar = 100 µm.

Supplemental figure 4
